# Supplementary material for: Survival Benefit of Renin-Angiotensin System Blockers in Critically Ill Cancer Patients: A Retrospective Study
Source: Cancers (Basel). 2023 Jun 14;15(12):3183. doi: 10.3390/cancers15123183 (PMC10296067; doi:10.3390/cancers15123183)
Supplement: Supplementary file 1 [file cancers-15-03183-s001.zip › cancers-2241520-supplementary.pdf]

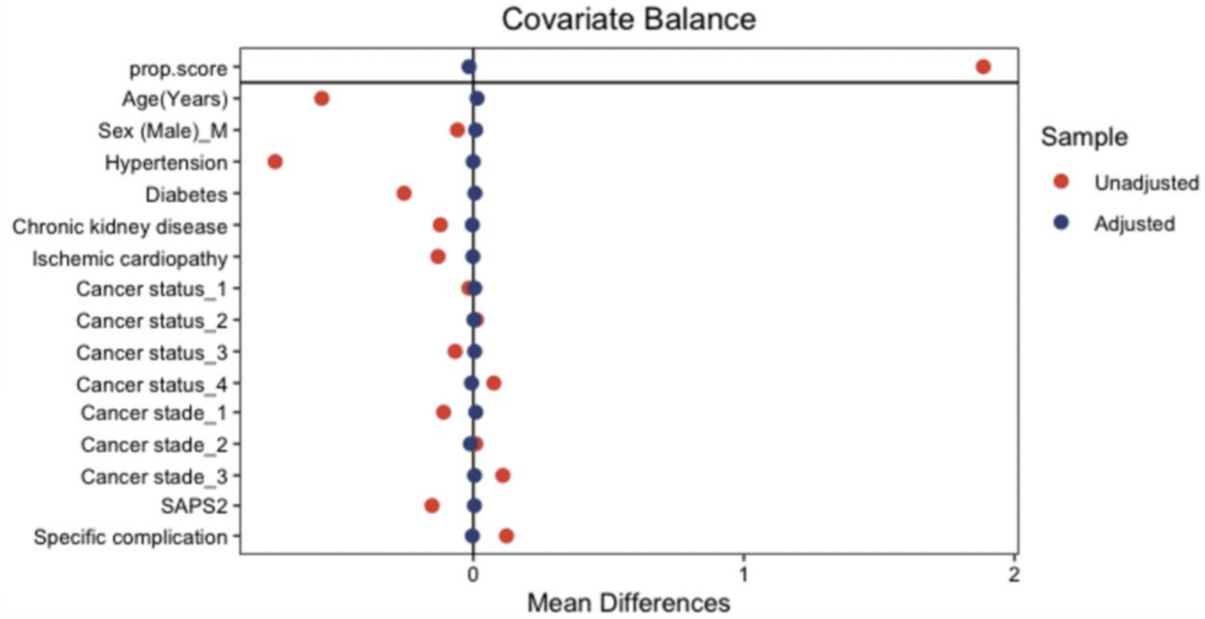


**Figure S1:** Balance of covariates in the adjusted and unadjusted cohort. Abbreviations: SAPS II: Simplified Acute Physiology Score II.
